# Institutional, humane, therapeutic: Towards an understanding of caregiver violence through third sector violence prevention discourse in Finland

**DOI:** 10.1111/1467-9566.13844

**Published:** 2024-09-26

**Authors:** Liina Sointu

**Affiliations:** ^1^ Faculty of Social Sciences Tampere University Tampere Finland

**Keywords:** care, discourse analysis, families, violence, violence prevention work

## Abstract

Violence in family caregiving, once a social taboo, is now emerging as a topic of scientific inquiry. Engaging with a non‐normative approach to care and critical reflection on research of abusive caregiver behaviour and elder abuse, this study argues that as caregiver violence is increasingly addressed in research, it is crucial to understand it as a complex issue within its social context. The study adds to this understanding by conducting a discourse analysis of violence prevention programmes in Finland, where third sector organisations have taken the initiative in addressing caregiver violence. Based on analysis of project materials, media texts and expert interviews related to two such violence prevention programmes, the study illuminates how caregiver violence is made intelligible through a combination of three kinds of understanding: institutional, humane and therapeutic. It is suggested that these understandings offer a way forward in thinking about the complex, sensitive topic of caregiver violence in sociological research on care.

## INTRODUCTION

Family care is a widespread phenomenon, even in the Nordic welfare states where public authorities are legally responsible for providing long‐term care (Kröger, 2019; Rostgaard et al., 2022). Family care emerged as an object of care policy in the United Kingdom and elsewhere in Europe starting in the 1970s (Heaton, 1999). Today, many European welfare states, Finland included, offer public support for family carers as an established part of their long‐term care systems (Kröger, 2019; Morgan & Zechner, 2022). The share of family care as part of long‐term care provision is likely to increase due to population trends and cutbacks in public long‐term care systems (Kröger, 2019).

The significance of families as providers of long‐term care calls attention to a sensitive issue which, although still a somewhat ‘hidden problem’ in the society (Isham et al., 2020) is also increasingly being addressed in research: that of violence in family care. The emergence of caregiver violence as an issue for the sociology of care is traced in the paper through two research literature, both in their own way highlighting the significance of acknowledging violence in care.

The first of these research literature asserts a non‐normative approach to care to better understand how violence emerges as part of care and care work (e.g., Cooper, 2007; Isham et al., 2020; Kelly, 2017; Simplican, 2015), but it has thus far mostly concentrated on violence experienced by caregivers, not on harm inflicted by caregivers on others. Another area of research has more directly addressed caregivers as perpetrators of violence (e.g., Beach et al., 2005; Cooper et al., 2008, 2010; Lafferty et al., 2016) and while this research area provides significant insight into the prevalence and individual risk factors for abusive caregiver behaviour and elder abuse, it has not thoroughly considered violence in the social and cultural context of family care (cf. Garnham & Bryant, 2017).

Based on critical reflection of these research literature, the present study argues that as the issue is increasingly addressed in research, it needs to be considered in all its complexity and within its social context. It is suggested that a discourse analysis of third‐sector violence prevention programmes may offer such an understanding. In Finland, third sector organisations, including family caregiver organisations, have taken the initiative in addressing caregiver violence through prevention programmes. Inspired by this development, this article examines the discourses of violence prevention to illuminate how caregiver violence becomes intelligible in and through violence prevention for family caregivers. Using expert interviews, materials produced by and media coverage of two such programmes as data, the paper sets out to answer the following research questions: how are violence and caregivers at risk of using violence constructed in the discourses of violence prevention? What kinds of wider cultural currents and discourses inform and underpin these understandings?

The paper begins by discussing in more detail the two literature and their critical reflection, through which caregiver violence emerges as a concern for the sociology of care: firstly, care researchers advocating for the acknowledgement of violence in the context of care and secondly, research on abusive caregiver behaviour and elder abuse and its critique. Then, Foucauldian discourse analysis as a theoretical starting point is explained. After that, the case of third sector violence prevention for caregivers in the Finnish long‐term care context is presented, followed by the data and methodology used in the study. The main findings—three understandings of violence—are then presented. Finally, the conclusion section discusses the significance of the results by concluding that together the three understandings—institutional, humane and therapeutic—offer a more comprehensive and contextualised understanding of caregiver violence which can be used to inform both research and societal approaches to the issue.

## TOWARDS AN UNDERSTANDING OF CAREGIVER'S VIOLENCE

For a long time, violence in the care‐giving context was a social taboo, but it is gradually becoming a concern for care policy and research (see e.g., Isham et al., 2020; Stewart, 2013, p. 73; Warburton‐Wynn, 2023). One strand of research through which caregiver violence emerges as a concern for the sociology of care is the pursuit towards a less normative approach to care. Such scholars as Cooper (2007), Kelly (2017) and Simplican (2015) take issues with implicitly conceiving care in terms of its virtues and ideals which make it difficult to address violence in care. According to this view, although no longer conceptualised as a ‘labour of love’ (Graham, 1983), care as a concept and research orientation still assumes an ethical stance in the world and in relation to others (Cooper, 2007; Simplican, 2015). Cooper (2007, p. 244) notes that ‘virtues, such as responsiveness, responsibility and attentiveness’ have long been regarded inherent in the concept of care.

Tensions, conflict and violence have not been completely absent from theorising care and care work (see e.g., Lee‐Treweek, 1994; Tronto, 1993, p. 135). For example, when conceptualising care as body work, Julia Twigg and colleagues note that social and health care ‘practitioners can experience sexism, racism, and other forms of abuse’ (2011, p. 175). In classic studies like Twigg's *Bathing* (2000, p. 2), it is suggested that care exist on a continuum ‘that at the one end represents the best sort of care and at the other verges on abuse’ such as ‘rough handling, denigrating language, sneering or nasty words’. For the most part, however, care receivers tend to be conceived as vulnerable and incapable of inflicting harm on others (Isham et al., 2020, p. 82; Simplican, 2015). Caregivers, on the other hand, tend to be perceived as affectionate and self‐sacrificing caterers to other people's needs according to gendered norms and expectations related to care. Against these conceptions, the idea of violence is unsettling (Kelly, 2017; Simplican, 2015).

That unsettlement, however, is not to be avoided or ignored; rather, it is a necessary aspect of broadening our understanding of care to include violence, as Christine Kelly argues (2017, p. 109). Kelly, like Cooper (2007, p. 244) and Simplican (2015), takes issue with idealised notions of care demanding a less normative approach to the topic. While not denying the virtues of care, they assert that normative assumptions hide other dimensions that are equally real, whether that is acknowledged or not (Kelly, 2017; Simplican, 2015).

In line with this demand, care researchers have focused on violence more directly through studying, for example, the structural and direct physical violence experienced and witnessed by care workers (Banerjee et al., 2012; Kelly, 2017), responding to violence with ‘caring self‐protection’ by care workers (Vaittinen, 2019), ‘bad care practices’ (Gil, 2021) and epistemic injustice faced by family carers who have experienced abusive behaviour by care receivers (Isham et al., 2020). These studies have each in their own ways contributed significantly to a view which is open to the ambivalence in care: how care involves conflict, hurt and violence—and how failure to acknowledge that results in epistemic injustice and suffering (Isham et al., 2020) and even death for the caregiver (Simplican, 2015).

Still, the focus has thus far mostly been on violence experienced by caregivers rather than on caregivers as perpetrators of violence. This may reflect a particular difficulty within care scholarship to attend to caregivers as perpetrators of violence when studying the experiences and practices of care. It may also reflect the shared pursuit of care scholars and activists for the recognition of the value of care work in society. From early on, care research contributed to the emergence and establishment of informal care discourse (Heaton, 1999, p. 764). Care has served as an idea and concept through which carers make claims for social justice (Barnes, 2006, pp. 135–136, 142). Against this background, it might be particularly difficult to draw attention to harm inflicted by family caregivers on care receivers.

Another strand of research, however, has directly addressed caregivers as perpetrators of violence showing that family caregivers do inflict harm on care receivers (e.g., Cooper et al., 2008; Pillemer & Suitor, 1992; Välimäki et al., 2020). Here the phenomenon has been conceptualised and studied for example, as abuse by family carers (Cooper et al., 2009), abusive behaviour (Cooper et al., 2010), abusive episodes (Steinsheim et al., 2022) and (potentially) harmful or abusive caregiver behaviour (e.g., Beach et al., 2005; Lafferty et al., 2016) and elder abuse (e.g., Cooper et al., 2008; Johannesen & LoGiudice, 2013; Lachs & Pillemer, 2004). Within this strand of research, physically abusive behaviour is considered to consist of, for example, rough handling, hitting, slapping, shaking or withholding food or medication, while examples of psychological behaviours are insulting, swearing, screaming or yelling, threatening to use physical force or threatening to cease providing care (e.g., Beach et al., 2005, p. 255; Lafferty et al., 2016, p. 705).

This research area has focused on assessing the prevalence of and risk factors for abusive caregiver behaviours and elder abuse. The share of caregivers who are estimated to engage in abusive or potentially abusive behaviours varies depending on the measurement and research contexts: based on studies made in the United Kingdom (Cooper et al., 2010) and in Ireland (Lafferty et al., 2016) approximately 30% of family caregivers had engaged in such behaviour, while in a Norwegian study almost 70% of caregivers reported committing at least one abusive episode within the last year (Steinsheim et al., 2022). Psychological and verbal abuse are most reported, while approximately 1%–10% of family caregivers report engaging in physical violence like rough handling, pinching or hitting (Lafferty et al., 2016; Steinsheim et al., 2022).

Neglect and failure to provide necessary care, whether intentionally or otherwise, also occur in family care. In a Norwegian study on abusive episodes in family care, approximately 6% of caregivers reported neglect (Steinsheim et al., 2022). It is often noted that self‐reported abusive behaviour is likely to be underestimated because the more serious the abuse, the less willing caregivers are to respond and—among those who do respond—the frequency and seriousness of abusive acts are likely to be underrated (Cooper et al., 2008, p. 158; Cooper et al., 2010, p. 140). In addition to prevalence, risk factors related to care receivers, caregivers and their relationships have been identified for both elder abuse (e.g., Lachs & Pillemer, 2004; Storey, 2020) and abusive caregiver behaviour (e.g., Beach et al., 2005; Pillemer & Suitor, 1992).

Studies on abusive caregiver behaviour and elder abuse advance the understanding of the extent of the issue and provide tools for the professional to identify high‐risk care‐giving situations. Alongside these advancements, there is however a need for critical reflection regarding how the issue of caregiver violence becomes understood through measuring prevalence and individual risk factors (cf. Garnham & Bryant, 2017). According to critical reflections of elder abuse research (Biggs & Haapala, 2010; Garnham & Bryant, 2017; Powell, 2012), the emphasis on measurement of prevalence and risk factors has given rise to a particular kind of discourse which justifies the authority of professional surveillance with little room for the voices of those directly affected by care (Garnham & Bryant, 2017; Powell, 2012). This results in a limited perspective on the issue which, according to Powell (2012, p. 3), may despite good intentions and genuine concern have negative consequences for caregivers and care receivers. These may include ‘increased moralism towards informal care and a move towards indirect monitoring’ (Powell, 2012, p. 7) without providing actual support for families with long‐term care needs.

The way in which caregiver violence emerges as a concern for research and policy is particularly significant in the current context of long‐term care. Due to austerity politics, cutbacks and reforms in long‐term care, as well as demographic trends, care responsibility is being shifted back to families in Finland and other European welfare states (Morgan & Zechner, 2022, p. 520). Family caregivers provide care, sometimes to multiple care receivers at the same time and often at the expense of their own health and wellbeing without enough public support. In this social context, it is essential that as caregiver violence is increasingly addressed in research, a contextual understanding of the issue is also developed (cf. Biggs & Haapala, 2010).

Based on critical reflection of these research literature the present study argues that as violence by caregivers is increasingly addressed in research, it needs to be considered in all its complexity and within its social context. The paper defines the issue as ‘caregiver violence’, aiming, on the one hand, to specifically address violence by caregivers. On the other hand, by employing the concept of violence rather than other available concepts like abuse or mistreatment, the paper conceives violence as culturally and socially embedded (Holmes et al., 2012; Husso et al., 2017). This implies that societal perceptions of acceptable and unacceptable behaviour are governed by prevailing ideas, norms and legislation (Husso et al., 2017). Methodologically, to study caregiver violence in its social context, the study draws from a discourse analysis of third sector violence prevention programmes in Finland. The paper is grounded on the idea that analysing texts and discourses may help to towards ‘more nuanced accounts of the complexities of violence in the social spaces of health care’ (Holmes et al., 2012, p. 8).

## APPROACHING CAREGIVER VIOLENCE THROUGH DISCOURSE ANALYSIS

Theoretically and methodologically, this article draws on the Foucauldian discourse analysis tradition to interpret violence prevention for caregivers. The idea is that through violence prevention programmes, a discourse has emerged through which caregiver violence is constructed as an object of knowledge in Finnish society as the issue has gained broader visibility in the media through the programmes (e.g., Heinonen, 2018; Trötschkes, 2018; Vasantola, 2021). Discourse is understood here as ‘a group of statements which provide language for talking about—a way of representing the knowledge about—a particular topic at a particular historical moment’ (Hall, 2001, p. 72). Within a discourse, there is consistency in what can be thought of and said about a phenomenon. In other words, a phenomenon is represented in a particular way, thus limiting and ruling out other ways of thinking (Hall, 2001, p. 73). Although discourse is an internally coherent system of knowledge, it is open and connected to available meanings and ways of thinking at any given historical moment (Hall, 1992, p. 292).

From these theoretical underpinnings, the preventive violence work discourse is understood to draw from prominent and available ways of thinking in the current society. One such is offered by the Finnish Institute for Health and Welfare and the World Health Organisation which provide institutionalised definitions for elder abuse and interpersonal violence (see Bildjuschkin et al., 2020). Another source for thinking about violence is offered by the ‘therapeutic turn’ which refers to the increasing influence of psychological and therapeutic rationalities and vocabularies in shaping the understanding of social phenomena (Illouz, 2008; Salmenniemi, 2022). Regarded as a prominent aspect of late capitalist culture, this phenomenon has been approached through concepts such as therapeutic culture and discourse (e.g., Illouz, 2008; Salmenniemi, 2022). Violence prevention work is informed by therapeutic discourse which provides tools, vocabularies and ideas originating from (popularised) psychology, self‐help books and ideas (e.g., self‐compassion), mindfulness and other concepts (Salmenniemi, 2022) with which to approach caregiver violence.

The aforementioned sources are an important part of the societal context in which violence prevention programmes operate in the Finnish third sector. The Foucauldian discourse tradition highlights the interconnectedness of language and practice: discourse is not ‘just words’ but connects to what is considered appropriate intervention, for example, and how this intervention is organised in practice (Heaton, 1999, p. 774). In other words, meaningful social practice is discursively formed (Hall, 1992, p. 291). In this way, analysing the discourse of violence prevention is indicative of not only how this phenomenon is ‘talked about’ but also about how it is addressed through practices, subjectivities and interventions. In addition, from the Foucauldian perspective, scientific research is intrinsically part of the discursive formations that pose social phenomena, such as caregiver violence, as ‘objects to be known’ (Garnham & Bryant, 2017, p. 53; Heaton, 1999). In this way, also the current study participates in formulating the discursive understanding of ‘caregiver violence’.

## VIOLENCE PREVENTION FOR FAMILY CAREGIVERS IN THE FINNISH THIRD SECTOR

The present study connects to two violence prevention programmes organised and carried out by third sector organisations, the only such programmes in Finland. In Finland, family care is supported by the public system: the *informal care allowance* combines a monetary benefit and entitlement to 3 days of caregiver leave per month covered by social services (Morgan & Zechner, 2022, p. 518). Altogether, 4.6% of Finnish people over 75 were provided long‐term care with the support of the informal care allowance in 2021 (Official Statistics Finland, 2022, p. 56). However, informal care by families is estimated to be a much more widespread phenomenon and likely to increase in the future due to cutbacks in long‐term care systems (Kröger, 2019). Regarding mistreatment and neglect, health and social care personnel have a legal responsibility to inform emergency social care and/or the police if they suspect abuse or neglect (Luoma et al., 2018).

In the Finnish context, third sector organisations, such as Suvanto Association, play an important role in raising awareness of elder abuse and providing support and guidance for older people who have experienced abuse through a national telephone helpline. However, family caregivers had not been explicitly targeted until the two violence programmes discussed in this article. Both were initiated, planned and structured by third sector organisations and financed by the Funding Centre for Social Welfare and Health Organisations. The organisations behind the programmes have an active role in initiating them as making the funding proposal requires initiative, engagement and expertise and not all projects receive funding because of a competitive application process. Both programmes were fixed term and intended for caregivers at risk of using violence towards care receivers.

The programmes informed caregivers about the opportunity to participate through their own organised activities, websites, social media, leaflets and articles and short news in local papers and Carers Finland newspaper and by giving presentations at events organised by carer organisations. Family caregivers independently initiated contact with the programmes via phone, e‐mail or in person. Participation in the programmes was completely voluntary and self‐initiated by caregivers who were invited to make contact if they had ‘difficult feelings’ and feared that they might hurt the person for whom they provided care for or had already done so.

The first programme, ‘Ei saa ravistaa’ (Do Not Shake, name translated by the author) (2017–2020) by Tampereen Seudun Omaishoitajat ry (Tampere Area Caregivers' Association) was a local initiative targeting family caregivers who had used or feared that they might use violence towards the family member for whom they provide care. This project involved various activities, including organised group sessions and individual consultations for family caregivers and presentations for health and social care professionals. The second programme, ‘Tunne voimavarasi’ (Know Your Strengths, name translated by the author) (2020–2023) was national in scope and was a collaboration of altogether four third‐sector organisations: Miina Sillanpään Säätiö (Miina Sillanpää Foundation), Kuntoutussäätiö (Rehabilitation Foundation), Maria Akatemia and Omaishoitajaliitto (Carers Finland). It focused on women aged 60 and up at risk of using violence against their care recipients. This project provided individual and group sessions for caregivers and workshops for professionals in seven locations in Finland. The programme developed an operational framework for peer groups to be freely accessible to family caregiver associations or anyone interested in the phenomenon. The model included a structured model of five group meetings for caregivers to be freely used by caregiver associations. An expert group comprising family caregivers actively participated in developing the operational model, which was subsequently tested by local caregiver associations.

Although the focus was on caregivers, both programmes also handed out materials and arranged events on a one‐time basis for wider audience and health and social care professionals to provide information about the specifics of abuse in family care situations, the forms and signs of abuse to be aware of and how to approach the issue when encountering family caregivers.

## METHODOLOGY

### Data

The data covers two violence prevention programmes consisting of materials produced by, media texts related to and four expert interviews related to these programmes. Altogether, there are approximately 400 pages of data. I was familiar with both projects and their premises before beginning to collect the data. I first came into contact with the ‘Do Not Shake’ project while visiting different caregiver associations to discuss the findings of my earlier research on family care (Sointu, 2016, 2018). At that time, the ‘Do Not Shake’ project had already begun and I was invited to follow its progress as part of a follow‐up group. This provided an opportunity to get to know the project and its approach, sparking my analytical interest in how the sensitive topic of violence was addressed. When the ‘Know Your Strengths’ project started, shortly after the ending of ‘Do Not Shake’, it became evident that a discourse on caregiver violence was emerging not only locally but also nationally and I began to plan the study that this article reports. I contacted the staff in the ‘Know Your Strengths’ project to express my interest and throughout its operation I occasionally attended events organised by the project to become familiar with the project and its approach.

By late 2022 and early 2023, both projects had ended and it was possible to systematically amass data by obtaining all the documents available on the project websites and other media. Key terms and project names were used to find all the media texts related to the programmes. At this point, I contacted both programmes to arrange interviews and conducted interviews with four of the five project staff members who worked with caregivers. The interviews covered the full span of the projects, focussing on the objectives, means and operations.

All these data sets were included to obtain the fullest possible sense of the discourse of violence prevention for caregivers in Finland. There are differences in how different texts were produced, for what purpose and by whom. Media texts were mostly written by journalists, while project staff served as experts about the phenomenon. Project materials were produced by project staff: they included psychoeducational and other materials to be used freely by caregiver organisations and social and health‐care professionals. These data were complemented by interviews with project staff members from both programmes. The interviews provided an opportunity to reflect that in greater detail. The interviews can be termed expert interviews (Gläser & Laudel, 2009, pp. 118–119); the interlocutors had special knowledge because of their role in the programmes.

Researching caregiver violence requires research ethical sensitivity. In carrying out the research, I have carefully followed the ethical guidelines for human participants (Finnish National Board on Research Integrity, 2019). Apart from the expert interviews, almost all the materials used as data in this study are publicly available. As the interviewees were already familiar with the topic, it was assumed that the topic would not cause distress for them. Therefore, according to the guidelines, no ethical pre‐evaluation was needed (Finnish National Board on Research Integrity, 2019, p. 20). The planning of the study involved thoughtful interview design so that the focus was on the programmes and their approach and not on individual caregiver cases. Because of media coverage of the programmes and the experts working in them, it was not possible to ensure the anonymity of either the projects or their staff. I consulted the interviewees to give them time to consider whether they would prefer the programmes to be anonymised as much as possible or mentioned by names—they preferred the latter. An important requirement of research ethics concerns representation and not causing harm to its subjects as individuals or as a group. In the present study, this connects to not only violence prevention programmes and their staff but also family caregivers more generally. Therefore, all wording and terms that describe the phenomenon and caregivers were carefully crafted.

### Analysis method

Drawing on the theoretical underpinnings presented, the analysis centres on how caregiver violence is constructed as an object of knowledge in and through violence prevention discourses. The specific research questions addressed are as follows: how is violence and caregivers who might use violence constructed in the discourses of violence prevention? and what kinds of wider cultural currents and discourses inform and underpin these understandings?

To answer these questions, four analytical questions were constructed, based on Stuart Hall's (2001, pp. 73–4) outline of elements important in studying discourse: (1) What is violence and what causes it? (*statements about the phenomenon*); (2) according to what kind of vocabularies and logics is violence talked about? (*rules or condition under which the phenomenon is considered*); (3) what is the caregiver who might use violence like? (*subjects embodying the discourse*) and (4) what should be done about violence, what measures should be taken and by whom? (*practices implicated in the discourse*).

As a result of organising and interpreting the data, three ways to conceive violence were identified: *institutional, humane* and *therapeutic*. These were not competing understandings, rather they complement one another. The first understanding served as a limit to the violence prevention work falling either outside or on the margins of preventive work, and the latter two formed the scope and ethos of actual preventive work. Of all the three, therapeutic understanding was the most prominent, covering the objectives and means of violence prevention work.

## RESULTS

### Violence as abuse and mistreatment

Within the institutional understanding, violence refers to severe abuse, neglect and mistreatment. This understanding is centred on the institutionalised, formal definitions of violence affirmed by organisations such as the World Health Organisation and the Finnish Institute for Health and Welfare (see Bildjuschkin et al., 2020). Within this understanding, violence is defined as abuse, mistreatment or neglect posing a serious risk to the health, wellbeing and even the life of the care receiver—or the caregiver.

Violence as severe abuse and mistreatment is perceived as requiring intervention from social welfare services and/or law enforcement. As such, severe abuse was ruled out of violence prevention work carried out in the programmes. However, severe abuse is in many ways present on the margins of violence prevention discourse. It is particularly visible in the materials intended for health and social care professionals. This is illustrated in the following quote:

The support and help provided in the project is based on the family caregivers' readiness to acknowledge their problematic behaviour and take responsibility for changing it. But professionals who work in social and health care organisations also encounter situations in which the family caregiver, for one reason or another, does not recognise the situation and is not ready to see a problem in his or her behaviour.

(Know Your Strengths, 2020a, p. 8)

Here, a distinction is made between prevention carried out in the programmes and other situations encountered by health and social care professionals with those caregivers who are not able to play an active role in ending their violent behaviour. The active participation of caregivers is a distinctive aspect of violence prevention, but professionals are called on to acknowledge that there are situations outside the scope of the preventive measures they can employ. While preventive work can be carried out in the third sector, the work of rebuilding an appropriate situation is to be carried out by public authorities like social workers and law enforcement.

Within this understanding, violence is conceived as something that can be objectively defined, observed and classified through formal criteria applied when assessing a situation from the outside. Accordingly, caregivers are objects of surveillance and intervention rather than agents in ending their violent behaviour. Violence is something for which professionals are responsible for taking action and they are provided with information about the forms of violence and signs of abuse and neglect. These are accompanied with concrete examples such as ‘signs of physical violence: fractures, bruises, wounds and burn injuries that the care receiver or the caregiver cannot explain believably’ (Know Your Strengths, 2020a, p. 9).

Both projects also offered guidance for the professionals and local family caregivers' associations about how to approach situations in which there is a suspicion about abuse. Guidance is provided about how to raise the issue in a conversation with caregivers and how to proceed if intervention is determined to be necessary. For the most urgent cases, emergency and crisis social services or law enforcement were identified as the correct authorities to contact. In addition, the programmes provided information and links to websites about making security plans both for care receiver and caregiver.

Violence as abuse and mistreatment is also implicitly present on the margins of preventive work as something that despite all best efforts, ultimately remains out of reach. This is partly due to the programmes' chosen emphasis on caregiver wellbeing and emotions, as illustrated in this quote:

It is unlikely that people who severely neglect or physically abuse a care receiver would reach out to our groups. I think it's highly unlikely, that they would apply to peer groups, because you know, “I want to enhance my own wellbeing and coping”. That doesn't really feel likely because at that point, you are so lost in yourself; I think at that point social welfare services are soon in the picture.

(Interview 3)

Here, it is explicitly noted that the approach through the caregivers' wellbeing would not work with those caregivers who use severe violence or who have neglected a care receiver. Yet the possibility of severe violence was not completely ruled out even among those caregivers who participated in the programmes, as it was stated that shame and stigma may prevent caregivers from disclosing abusive acts in peer groups. In this way, abuse and mistreatment inhabit the margins of preventive work:

Marjatta has not yet attacked Erkki. But she is afraid that one day it might happen: “We will continue to have situations in which this may happen”. The coordinator in the project “Do Not Shake” says that they have not yet encountered direct violence in their peer groups. (…) This is not, however, necessarily the whole truth. It might be that violence is used, but it is too difficult to talk about it.

(Heinonen, 2018, p. 34)

Here violence is referred to as ‘attacking’ and ‘direct violence’. It is acknowledged that violence may be used but remain undisclosed. In this way, violence as severe physical abuse is implicitly part of violence prevention discourse, although it is in many ways understood as beyond the scope of preventive measures as such. Thus, severe mistreatment and neglect defined the scope and limits of violence prevention work. Severe abuse was conceived, implicitly or explicitly, as a situation in the future to be avoided and prevented or as already taking place and requiring surveillance and intervention on the part of health and social care professionals.

### Violence as a shared human condition

Another understanding reflects a humane approach to violence. Within this understanding, violence is viewed as a human condition. By approaching violence through shared humanity, it is possible to address an issue that has the potential to induce intense shame—and thus remain hidden. The central idea here is that anyone may commit a violent act, as illustrated here:

It is important for me to stress the human side of this, to understand that we all have the potential to use violence. It's not like that there are those violent persons and we non‐violent persons. It's a universal phenomenon shared by all human beings, and it just takes courage to see that.

(Interview 3)

It is asserted here that because violence is a human condition, people cannot be divided into violent and non‐violent: it is not a matter of whether one has a capacity for violence but about whether a person is willing to acknowledge that capacity. In this way, violence is not perceived as pathological or criminal but rather as part of human life.

In addition to being part of the human condition, violence is conceived here through its everyday nature. It is stressed that violence is nothing extraordinary or drastic: ‘I want to emphasise just how mundane it can be’ (Interview 2). Within this understanding, there is also space to consider whether something in the caregiver's conduct in relation to the care receiver is or is not in fact violence. Unlike the institutional understanding, there are no ready‐made criteria to determine what violence consists of; rather, violence has to do with the context as something that emerges and receives its meaning as part of everyday human interaction. An important context is the care receiver's illness, which can set new expectations for appropriate conduct. It may be that certain behaviours, especially if repeated, could be termed violent in the care context even though that was not previously the case:

That everyday situations can lead to it, that it does not have to be so dramatic and unusual that your emotions boil up. (…) A person might not think that it is mistreatment if you talk very nastily to the other person in a more vulnerable position: how cruel it can be, how much you can hurt with those words.

(Interview 2)

These considerations take place in the context of the changing power dynamics from an intimate couple's relationship to one where the care receiver depends on the caregiver for emotional security among other things. For caregivers to be able to reflect on their own actions, they need an environment where they feel safe. One expert described how in encounters with caregivers, they tried to convey the message ‘that we're all human beings, and create this immense acceptance, that kind of atmosphere’ (Interview 1). It was emphasised that caregivers are to be accepted as human beings, even when their violent acts are not acceptable: ‘We always stressed that you are still a good human being; you just must start doing something about this’ (Interview 2).

As a shared human condition, the capacity for violence and non‐violence unites all human beings: caregivers, professionals and those doing violence prevention work. Caregivers who are at risk of using violence are deemed as ‘normal’ human beings as opposed to ‘pathological’ or somehow ‘sick’. It was viewed as important that those who work with caregivers had done ‘the inner work’ with their own experiences of violence. Although distinct in its emphasis on shared humanity and acceptance, the humane understanding also overlaps with the therapeutic understanding through its emphasis on that inner work.

### Violence as violent behaviour emerging from repressed emotions

The most prominent understanding of violence in violence prevention work can be characterised as therapeutic. This understanding permeates the objectives, means and desired outcomes of both programmes, reflecting the emphasis of therapeutic culture on holistic wellbeing, personal growth and self‐care (Salmenniemi, 2022). Here, the origins of violence are located within the caregiver's psyche and the overwhelming circumstances of care responsibility. Violence arises from emotions, especially aggression:

If one has had to repress challenging emotions in one's life, they may burst out as uncontrollable behaviour in stressful circumstances that hurts, frightens or in other ways inflicts harm to oneself or another person. This kind of behaviour is called mistreatment or violence. Aggression can serve as a life force, but also as a destructive force.

(Know Your Strengths, 2021, p. 17)

Within this understanding, violence is predominantly talked about as ‘violent behaviour’, referring to actions which the caregiver cannot control and which could be harmful for the care receiver. This kind of behaviour is associated with emotions deemed as ‘difficult’, particularly anger. It is central, however, that emotion and behaviour be separated from each other. Although violent behaviour is understood as emerging from repressed aggression and other underlying emotions, those emotions as such are not deemed as violence. This is stressed in the programmes and is aptly summed up by the motto of the ‘Do Not Shake’ project: ‘All emotions are allowed, but shaking is not’.

Uncontrollable anger, overwhelming situations and exhaustion are conceived to potentially evolve into violent acts or behaviours. Examples mentioned by the interviewees include over‐medication, locking the care receiver in a room and leaving them without attention or not allowing the care receiver to watch TV as a punishment for something that happened earlier. In addition, self‐harm is explicitly acknowledged: violent behaviour may not only be directed towards care receivers but also to caregivers themselves. Examples include ignoring caregiver's own needs and not allowing oneself to experience joy or respite.

Within this understanding, the underlying reason for violence is perceived to be an overwhelming life situation and/or an unbalanced emotional life caused by an individual's psychological history and compounded by the pressures of care‐giving. The objectives and means of violence prevention work reflect these ideas:

The objective of the peer group is that participants learn to recognise their strengths and find ways to cope in their daily lives. Caregiver fatigue is the biggest risk factor for mistreatment in the care relationship. Therefore, strengthening the capacity to recognise one's own needs and setting limits as part of the support for one's own wellbeing are central ways to prevent mistreatment whether it is directed towards the care receiver or the caregiver's own self.

(Know Your Strengths, 2022)

Setting limits and finding safe ways to express anger are understood to be central to preventing violence: if caregivers learn to set limits and take care of themselves, they may not become exhausted by their caring duties and, thus, will not engage in violent behaviour. The difficulties in limit setting are understood not only in connection to care needs but also to caregivers' childhoods, social roles and relationships before the present situation. Although the therapeutic understanding places a great deal of emphasis on the individual psyche, emotions and emotional skills, caregivers are simultaneously perceived as part of a society which expects women to suppress their own needs for the sake of others as illustrated in a video about women's violence: ‘there lives a wounded girl within a woman who acts violently’ (Know Your Strengths, 2020b, 1:27).

It is emphasised even within the therapeutic understanding that caregivers are responsible for their own conduct. Commitment to ending abusive behaviour begins with acknowledging and admitting such behaviour through some form of confession, as illustrated here:

Waking up to one's own violence tends to evoke guilt, shame and anguish. Taking responsibility for one's own violence and admitting one's violent behaviour are the most important and most difficult steps in the pursuit of ending violent behaviour.

(Know Your Strengths, 2020a, p. 11)

To confess, caregivers need to be able to use concrete names for their acts of violence. Accordingly, going through unique situations and identifying and naming specific acts was an important aspect of individual consultations in the programmes. Although confessions were not expected in the peer groups as such, caregivers were provided with concrete examples of different forms of violence as part of the group activities. Here, the therapeutic understanding is clearly entangled with the institutional understanding of violence.

The therapeutic understanding of violence implies an active involvement of the caregiver in the process of ending his or her violent behaviour. Acknowledging one's own violent behaviour, even towards oneself, and committing to take better care of oneself is conceived as a crucial means to prevent violence. Additionally, caregivers are encouraged to learn about and process emotions and practice their emotional skills. It is considered extremely important that caregivers take responsibility for their own actions, but this is something that cannot be enforced from the outside; rather, it requires self‐exploration and self‐compassion.

## CONCLUDING DISCUSSION

The present study set out to examine how violence—and caregivers at risk of using violence—are constructed as objects of knowledge through third sector violence prevention discourse. As a result, three main understandings of violence were identified: institutional, humane and therapeutic. First, through an institutional understanding, caregiver violence is constructed as serious abuse and neglect that lie mostly beyond the scope or on the margins of preventive work. Family carers are constructed as objects of surveillance and intervention by health and social care professionals. According to this understanding, caregiver violence is deemed a severe social welfare or even a criminal issue to be intervened by public authorities and professionals.

Second, through a humane understanding, violence is understood to be part of the human condition and perceived through its everydayness: there is nothing extraordinary about violence. Whether or not an act is violent depends on the circumstances, such as an intimate relationship becoming a care relationship and bringing with it new expectations for caregiver conduct. Caregivers at risk of using violence are understood as human beings, just like anyone else.

Third, in the therapeutic understanding violence is constructed as uncontrollable, problematic behaviour arising from exhaustion and/or inadequate emotion management. Caregivers are perceived as subjects with needs and emotions and their wellbeing is a relevant concern. The central way to prevent violence by caregivers is to enhance their wellbeing through self‐care.

To conclude, it is asserted in this study that as violence is gradually becoming addressed in public and academic discourses, these discourses should be informed by an understanding of the complexity of the issue in the context of long‐term care. As long‐term care policies in Finland and in other European create pressure for families to provide care (Hansen & Dahl, 2021; Kröger, 2019), there clearly is a pressing need to ensure the safety and wellbeing of both care receivers and caregivers living at home with long‐term care needs. Accordingly, research on abusive caregiver behaviour has emerged to accompany already established research area of elder abuse. Research on the prevalence and risk factor for abusive caregiver behaviour (e.g., Beach et al., 2005; Cooper et al., 2008; Lafferty et al., 2016) have been helpful in identifying the problem and indicating its breadth and the risk factors to be aware of.

However, increased screening and monitoring by professionals, often suggested by these studies (e.g., Lafferty et al., 2016, p. 706), also warrant critical reflection. While there are situations in which intervention by social and health care professionals and law enforcement is crucial to protect the safety of both care receivers and caregivers (Luoma et al., 2018), institutional understanding captures only one, albeit significant, dimension of violence in care‐giving settings. Increased surveillance and detailed code of conduct, without appropriate support services, may end up creating more pressure for already overburdened carers particularly if approached from top‐down and standardised ways (cf. Powell, 2012; Winch, 2006).

As highlighted by the humane understanding of violence outlined in this article, there is a need for a discursive space where caregivers can reflect on the complexities of their everyday lives without moral judgement. This space is needed for caregivers to discern whether a previously non‐problematic behaviour might be considered violent within the context of a care‐giving relationship, where power dynamics have been altered by care needs and responsibilities (cf. Biggs & Haapala, 2010, pp. 177–178). The idea of a caregiver inflicting harm on the care receiver quickly raises moral judgement, potentially contributing to the concealment of violence. Acknowledging ambiguity as part of care does not mean that abusive actions should be normalised and justified for their everydayness. Instead, it recognises that family caregivers must navigate care as a moral practice in challenging circumstances (Pickard, 2010).

Therapeutic understanding of violence highlights the significance of family caregiver's wellbeing holistically and in connection to emotions. Through this understanding, it becomes possible to see the caregiver as someone with needs—and whether these needs are fulfiled is connected to the quality of care. Again, unfulfilled needs of the caregiver do not justify violence, but they should be taken into account in the current context of long‐term care which creates pressure on families to provide care (Hansen & Dahl, 2021; Kröger, 2019). Sufficient support for family care and the possibility to disengage from care responsibility temporarily and/or permanently are highlighted here as significant in ensuring the safety and wellbeing of both care receivers and caregivers.

While the results are based on a discourse analysis of a Finnish case, it is suggested that these three understandings are useful for considering the issue beyond the Finnish context. There are, however, limitations to be considered. The data include only expert interviews, project materials and media texts related to violence prevention programmes; there are no participant observations nor interviews with family caregivers. For ethical and practical reasons, it was determined that this study would be limited to those types of data. Therefore, it does not provide insights into the actual practices that have taken place as part of violence prevention work. Similarly, this article does not concern the lived experiences of either caregivers or care receivers and thus does not indicate anything about how the participants experience and negotiate these practices and understandings.

Despite these limitations, this article contributes to the sociological understanding of caregiver violence by illuminating how the issue has emerged as an object of knowledge through third‐sector violence prevention discourse in Finland. This study suggests that there is no single way to conceive this issue. It proposes that the three understandings offer a nuanced perspective on caregiver violence, providing a way forward in addressing this complex and sensitive topic in sociological research. Further research from other contexts and perspectives is needed to achieve a more comprehensive understanding of violence within care relationships and society.

## AUTHOR CONTRIBUTIONS

**Liina Sointu**: Conceptualisation (lead); data curation (lead); formal analysis (lead); investigation (lead); methodology (lead); project administration (lead); writing—original draft (lead); writing—review & editing (lead).

## CONFLICT OF INTEREST STATEMENT

The author declares that they have no conflict of interest.

## ETHICS STATEMENT

Although the topic—caregiver violence—can be considered sensitive, the interviewees were already familiar with the topic through their work and therefore it was assumed that the topic would not cause distress for them. Therefore, according to the ethical guidelines of Finnish National Board on Research Integrity, no ethical pre‐evaluation was required.

## PATIENT CONSENT STATEMENT

Not applicable.

## PERMISSION TO REPRODUCE MATERIAL FROM OTHER SOURCES

Not applicable.

## Data Availability

The data that support the findings of this study are partly openly available online in Finnish. For more detailed information on accessing these online sources, interested parties may contact the corresponding author. Due to confidentiality agreements, expert interviews are not shared.
